# Retroviral transduction of peptide stimulated t cells can generate dual t cell receptor-expressing (bifunctional) t cells reactive with two defined antigens

**DOI:** 10.1186/1479-5876-2-42

**Published:** 2004-12-08

**Authors:** Alexander Langerman, Glenda G Callender, Michael I Nishimura

**Affiliations:** 1 Surgical Oncology Laboratory, Department of Surgery, Section of General Surgery, University of Chicago, Chicago IL USA; 2 Pritzker School of Medicine, University of Chicago, Chicago IL USA

## Abstract

**Background:**

Tumors and viruses have developed many mechanisms to evade the immune system, including down-regulation of target antigens and MHC molecules. These immune escape mechanisms may be able to be circumvented by adoptively transferring T cells engineered to express two different T cell receptors, each specific for a different antigen or MHC restriction molecule.

**Methods:**

PBMC from the blood of normal healthy donors were stimulated for three days with an antigenic peptide from cytomegalovirus (CMV) pp65. These CMV reactive cultures were transduced with a encoding the TIL 5 T cell receptor (TCR) that mediates recognition of the dominant epitope of the melanoma antigen MART-1. Following selection for transduced cells, the cultures were evaluated for recognition of CMV pp65 and MART-1 expressing targets.

**Results:**

We were able to rapidly create bifunctional T cells capable of recognizing both CMV pp65 and MART-1 using a combination of HLA-A2 tetramer staining and intracellular staining for interferon-γ. These bifunctional T cells were sensitive to very low levels of antigen, recognize MART-1^+ ^tumor cells, and maintained their bifunctionality for over 40 days in culture.

**Conclusion:**

Bifunctional T cells can be engineered by transducing short term peptide stimulated T cell cultures. These bifunctional T cells may be more effective in treating patients with cancer or chronic virus infections because they would reduce the possibility of disease progression due to antigen and/or MHC loss variants.

## Background

It has long been established that tumors and viruses have multiple mechanisms for evading the immune system including the inhibition of T cell function through the release of inhibitory cytokines and factors [1,2], down-regulation of MHC molecules [2,3] and the spontaneous generation of antigen-loss variants [1-3]. In latter case, despite the loss of a single antigen on a tumor or virus-infected cell, there can remain functional HLA class I molecules and multiple antigens that can serve as targets for immune destruction. Therefore, immunotherapy strategies which target multiple antigens and/or multiple HLA class I molecules may be more effective than therapies targeting single antigens presented by a single HLA class I molecule.

We and others have shown that it is possible to use retroviral vectors encoding TCRs isolated from tumor- or virus-reactive T cell clones to engineer human T cells to recognize any antigen [4]. While every T cell that is transduced to express a second TCR expresses its own TCR capable of recognizing some antigen, it has only recently been shown that "bifunctional" T cells capable of recognizing two known antigens can be generated [5]. Using this technology, it may be possible to treat patients with T cells bearing two functional T cell receptors (TCRs) with each TCR being specific for a different tumor-associated antigen (TAA) or viral antigen restricted by one or more HLA molecule. These bifunctional T cells would retain effectiveness against single antigen-loss variants or HLA loss variants and may have improved efficacy over monospecific T cells for the treatment of tumors or viruses.

In the current study, we show that it is possible to rapidly generate T cell populations containing T cells reactive with two defined antigens, CMV pp65 and MART-1. These T cell cultures are highly avid for both antigens and retain their reactivity for at least six weeks. More importantly, this methodology could easily be adapted to closed culture systems making it more attractive for use in clinical trials.

## Methods

### Tumor Cell Lines

All melanoma and renal cell carcinoma cell lines used in this study were established from surgical specimens obtained from cancer patients undergoing immunotherapy at the Surgery Branch, National Cancer Institute. Melanoma cell lines 624 MEL (HLA-A2^+^, MART-1^+^), 624-28 MEL (HLA-A2^-^, MART-1^+^), 1300 MEL (HLA-A2^+^, MART-1^+^), and SK23 MEL (HLA-A2^+^, MART-1^+^) and renal cell carcinoma cell line UOK131 (HLA-A2^+^, MART-1^-^) were maintained complete medium (CM) which consisted of RPMI 1640 medium (Life Technologies, Gaithersburg, MD) supplemented with 10% heat inactivated fetal bovine serum (Life Technologies), and penicillin (100 U/ml)/streptomycin (100 μg/ml)/L-glutamine (2.92 mg/ml)(Life Technologies) as described [6]. The PG13 A7 retroviral producer cell line, the source of the TIL 5 TCR retrovirus used in this study, has been described elsewhere [7]. Retroviral production by PG13 A7 cells was carried out using an optimized protocol described by Lamers *et al *[8] in T175 flasks at 32°C in CM. T2 cells and COS A2 were maintained in CM as described [6].

### T Cells

R6C12 is an HLA-A2 restricted, gp100:209–217 reactive CTL clone that was isolated from the peripheral blood of a melanoma patient vaccinated with the gp100:209–217 210M peptide at the Surgery Branch, National Cancer Institute. R6C12 cells were expanded using 10 ng/ml anti-CD3 mAb (Ortho Biotech, Raritan, NJ) and 300 IU/mL recombinant human IL-2 (rhIL-2) (Chiron, Berkeley, CA) in T cell medium (TCM) which consisted of RPMI 1640 supplemented with 10% pooled human AB serum (Valley Biochemical, Winchester VA), HEPES (Life Technologies), 2-mercaptoethanol (Life Technologies), penicillin (100 U/ml)/streptomycin (100 μg/ml)/L-glutamine (2.92 mg/ml)(Life Technologies) as described [9]. Peripheral blood mononuclear cells (PBMC) obtained from leukapheresis of healthy donors were used as a source of T cells for establishing CMV pp65 peptide stimulated T cell cultures and feeders for T cell expansion were purchased from BRT Laboratories (Baltimore, MD).

### Peptides

All peptides used in this study were purchased from Synthetic Biomolecules (San Diego, CA). Peptides used were MART-1:27–35 (AAGIGILTV), Influenza M1:58–66 (GILGFVFTL), CMV pp65:495–503 (NLVPMVATV), or gp100:209–217 (ITDQVPFSV). Each peptide was maintained as a concentrated stock (2–5 mg/ml) in 100% DMSO (Sigma, St. Louis, MO) and diluted in the appropriate medium prior to immediate use.

### CMV pp65 Expressing Targets

Given that it is technically difficult to obtain CMV infected targets for immunologic assays, COS A2 cells were engineered to express a mini-gene encoding the CMV pp65:495–503 peptide epitope (COS A2 CMV). A CMV minigene was constructed using complementary synthetic oligonucleotide primers (sense primer: 5'-GGCCCGCGCAGGCAGCATGAACCTGGTGCCCATGGTGGCTACGGTTTAGTGA-3', anti-sense primer: 5'-GGCCTCACTAAACCGTAGCCACCATGGGCACCAGGTTCATGCTGCCTGCGCG-3', Integrated DNA Technologies, Coralville, IA) that encoded the CMV pp65:495–503 peptide epitope with an ATG translation initiation codon, a Kozak consensus sequence [10] and Not I compatible "sticky ends" to facilitate insertion into the Not I site of the SAMEN CMV/SRα retrovirus. Equal molar amounts of each synthetic oligonucleotude were mixed and ligated into the SAMEN CMV/SRa retrovirus using a rapid ligation protocol and transformed into DH5α competent *E. coli *cells (Life Technologies) as described [11]. Recombinant clones were sequenced to insure proper orientation and retroviral supernatants were produced by cotransiently transfecting 293GP cells with plasmids encoding the retroviral backbone and the vesicular stomatitis virus envelope as described [11]. COS A2 CMV cells were generated by culturing COS A2 cells overnight with retroviral supernatants supplemented with 8 μg/ml polybrene (Sigma).

### Peptide Stimulation and Transduction of PBMC

PBMC from healthy donors were stimulated *in vitro *with 5 μg/ml of CMV pp65:495–503 peptide in TCM containing 300 IU/mL IL-2 for 3 days. T cell cultures were then transduced using a modified Retronectin (TaKaRa, Otsu, Japan) protocol with the A7 retrovirus as follows: 24-well plates were coated with Retronectin then were preloaded with retrovirus according to the manufacturer's instructions. 2.6 × 10^6 ^T cells were added to each well in 1.3 ml (2 × 10^6 ^cells/ml) of A7 retroviral supernantant supplemented with 300 IU/ml rhIL-2 and the plates were centrifuged for 90 min at 1000 g. The next day the medium was replaced with fresh A7 retroviral supernatant and the centrifugation was repeated. The cells were rested for 24 hours and then transduced cells were selected in 1 mg/ml of G418 (Research Products International, Mt. Prospect, IL) for five days. Cultures were assayed for antigen reactivity, cyropreserved, and/or expanded for additional assays.

### Transduction of T Cell Clones

T cell clone R6C12 was cultured at 2 × 10^6 ^cells/ml in TCM supplemented with 300 IU/ml rhIL-2, and 2 μg/ml anti-CD28 mAb (Becton, Dickenson, and Company, Franklin Lakes, NJ) in 24 well tissue culture plates pre-coated overnight with 10 μg of anti-CD3 mAb (Ortho Biotech, Bridgewater, NJ) for three days prior to transduction. Transduction of R6C12 was carried out as described above for CMV peptide stimulated T cell cultures except 2 μg/ml anti-CD28 mAb was added to the medium and 10 μg anti-CD3 mAb was bound to the culture plates in addition to Retronectin.

### Antigen Recognition Assays

The antigen reactivity of each T cell culture (TIL 5 TCR transduced and untransduced) was assayed for MART-1:27–35 and CMV pp65:495–503 or gp100:209–217 reactivity in interferon-γ release assays. 5 × 10^4 ^T cells were cocultured in a 1:1 ratio overnight in 0.2 ml of CM in duplicate individual wells of a 96-well plate with a panel of stimulators that included T2 cells loaded with 5 μg/mL MART-1:27–35, Influenza M1:58–66, CMV pp65:495–503, or gp100:209–217 peptide and a panel of tumor cells. The amount of interferon-γ released was measured by ELISA as described [6].

### Intracellular Cytokine Release Assay and Tetramer Staining

The existence of CMV pp65:495–503/MART-1:27–35 reactive bifunctional T cells was determined by first staining T cells for intracellular interferon-γ production following coculture with HLA-A2^+ ^MART-1^+ ^stimulator cells followed by fluorescence staining with HLA-A2/CMV pp65:495–503 tetramers. 1 × 10^5 ^T cells were cocultured in a 1:1 ratio peptide loaded T2 cells or tumor cells for five hours in CM supplemented with 10 μg/ml brefeldin-A. Cells were then collected and stained with PE-conjugated HLA-A2/CMV pp65:495503 tetramers (Beckman Coulter Immunomics, San Diego, CA), fixed in 1% paraformaldehyde (Sigma), permeabilized using 0.5% saponin (Sigma), and then stained with FITC-conjugated anti-interferon-γ (Biosource International, Camarillo, CA). Relative log fluorescence of 10^4 ^live cells was measured by flow cytometry using a FACS Scan flow cytometer (BD Biosciences, Mountain View, CA).

## Results

### Recognition of Peptides and Tumor Cells by TIL 5 TCR-transduced CMV peptide stimulated T cells

**Figure 1:**
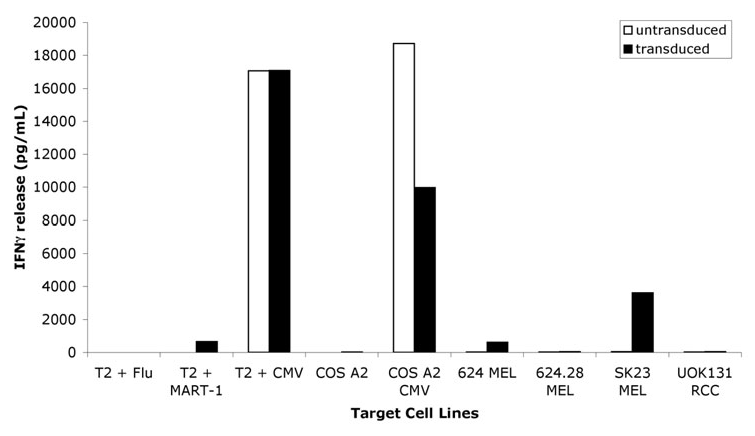
**Recognition of peptides and tumor cells by TIL 5 TCR-transduced CMV-stimulated T cells. **TIL 5 TCR-transduced and untransduced CMV-stimulated bulk cultures were cocultured for 24 hours in a 1:1 ratio with T2 cells loaded with 5 μg/ml of peptide, COS A2 cells with or without a CMV minigene, or A2^+^, MART-1^+ ^tumor cells (SK23 MEL, 624 MEL), A2^-^, MART-1^+ ^tumor cells (624.28 MEL), or A2^+^, MART-1^- ^tumor cells (UOK131 RCC). Supernatants were collected and the amount of interferon-γ released was measured using ELISA. Values are the average of triplicate wells.

**Figure 2:**
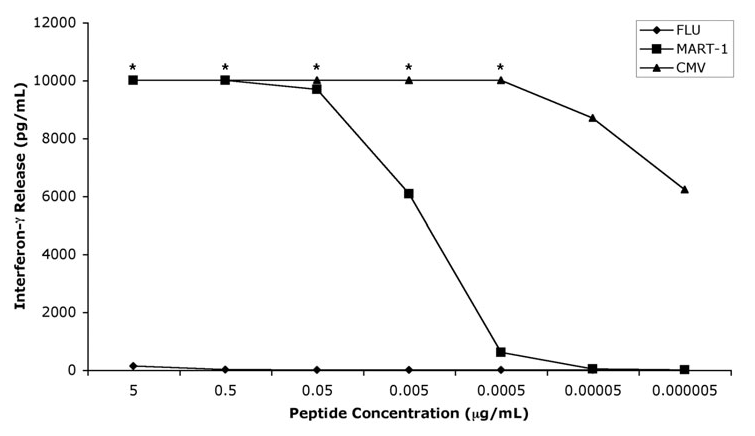
**Sensitivity of bifunctional cultures to low levels of recognized antigens. **TIL 5 TCR-transduced cultures were cocultured for 24 hours in a 1:1 ratio with T2 cells loaded with decreasing concentrations of peptide. Supernatants were collected and the amount of interferon-γ released was measured using ELISA. Values are the average of triplicate wells. Asterisk (*) indicates value was greater than maximum point on standard curve.

**Figure 3:**
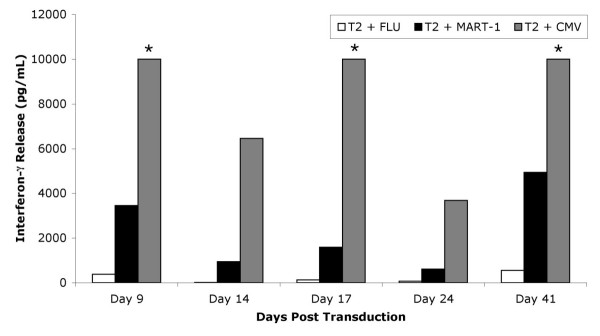
**Long term maintenance of bifunctionality in culture. **TIL 5 TCR-transduced cultures were cocultured for 24 hours in a 1:1 ratio with T2 cells pulsed with 5 μg/ml peptide at varying time points beyond transduction. Supernatants were collected and the amount of interferon-γ released was measured using ELISA. Values are the average of triplicate wells. Asterisk (*) indicates value was greater than maximum point on standard curve.

Bifunctional T cells reactive with CMV and MART-1 were engineered by first stimulating donor PBMC with CMV pp65:495–503 peptide for three days then transducing the T cell cultures with a retrovirus encoding a TCR specific for MART-1:27–35 presented by HLA-A2. After five days of selection in G418, the T cells were assayed for reactivity against the CMV pp65:495–503 and MART-1:27–35 antigens in interferon-γ release assays. Significant amounts of interferon-γ were released when the TIL 5 TCR-transduced CMV peptide stimulated T cells were cocultured with CMV pp65:495–503 or MART-1:27–35 peptide-loaded T2 cells, COS cells engineered to express HLA-A2 with a CMV pp65:495–503 mini-gene, or HLA-A2^+ ^MART-1^+ ^tumor cells (Figure 1). These cells did not release interferon-γ when stimulated with T2 cells loaded with Flu M1:58–66 peptide, COS A2 (MART-1^- ^CMV^-^), 624-28 MEL (HLA-A2^- ^MART-1^+ ^CMV^-^), or RCC UOK131 (HLA-A2^+ ^MART-1^- ^CMV^-^) cells. Untransduced CMV peptide stimulated T cells only released interferon-γ when stimulated with CMV pp65:495–503 peptide loaded T2 or COS HLA-A2^+ ^CMV^+ ^cells. These cultures were extremely sensitive to antigen stimulation since significant amounts of interferon-γ were released when stimulated with T2 cells loaded with 5 × 10^-4 ^μg/mL MART-1:27–35 peptide and <5 × 10^-7 ^μg/mL CMV pp65:495–503 peptide (Figure 2). These bulk cultures also continued to be reactive to both antigens more than 40 days post transduction (Figure 3). These results indicate that three day peptide stimulated PBMC cultures can be activated *in vitro *for efficient retroviral transduction. Furthermore, the antigen reactivity of these T cells is consistent with bifunctional T cells capable of recognizing both CMV and MART-1.

### Antigen Recognition by CMV-tetramer positive T cells

While the antigen reactivity of our T cell cultures was consistent with us having engineered bifunctional T cells, it was necessary to confirm that individual T cells possess the capability to recognize both CMV pp65 and MART-1. To confirm that we had engineered bifunctional cells, each T cell culture was stained with HLA-A2/CMV pp65:495–503 tetramers for anti-CMV reactivity and with intracellular anti-interferon-γ monoclonal antibodies following stimulation with HLA-A2^+ ^MART-1^+ ^cells for anti-MART-1 reactivity. It should be noted that the reciprocal experiment, staining with HLA-A2/MART-1:27–35 tetramers and intracellular anti-interferon-γ staining following CMV pp65:495–503 peptide stimulation could not be performed since TIL 5 TCR expressing cells do not bind HLA-A2/MART-1:27–35 tetramers (unpublished).

**Figure 4:**
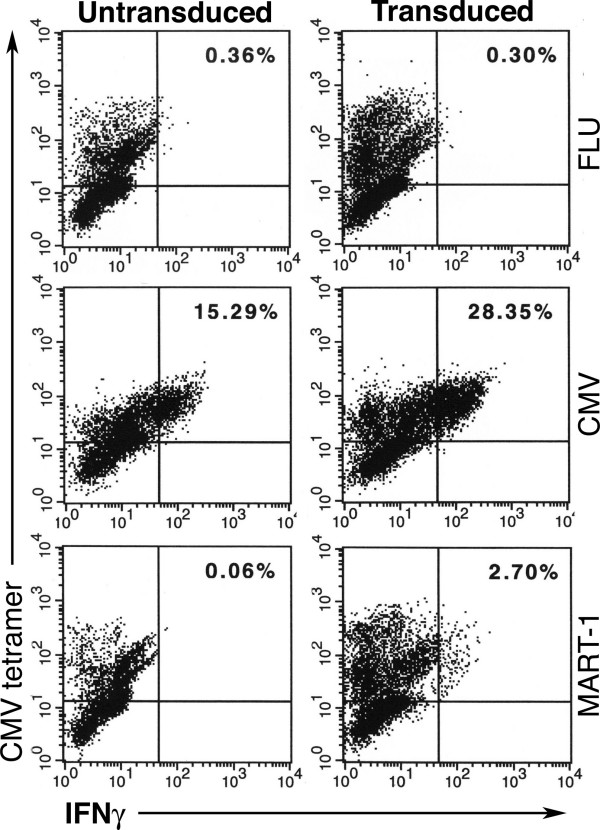
**Peptide recognition by CMV tetramer^+ ^T cells. **TIL 5 TCR-transduced and untransduced CMV-stimulated bulk cultures were cocultured in the presence of 10 μg/ml brefeldin A for 5 hours in a 1:1 ratio with T2 cells pulsed with 5 μg/ml of peptide. Cells were then collected, stained with PE-conjugated HLA-A2 MHC CMV tetramer, fixed, permeabilized, and stained with FITC-conjugated anti-IFN-γ mAb. Samples were analysed using two-color flow cytometry. The percentage of dual positive staining cells (upper right quadrant) is as indicated.

Cells that were double stained with tetramers and for intracellular anti-interferon-γ were considered to be reactive with both antigens and therefore bifunctional. As shown in Figure 4, 2.7% of the TIL 5 TCR-transduced T cells were double stained following stimulation with MART-1:27–35 loaded T2 cells compared to 0.06% of the untransduced T cells. When stimulated with CMV pp65:495–503 peptide loaded cells, 28.35% of the TIL 5 TCR-transduced T cells were double stained. These results are representative of multiple cultures which routinely have approximately 10% of the CMV reactive T cells also recognizing MART-1. These results confirm that bifunctional T cells can be obtained by transducing three day peptide stimulated PBMC cultures with retroviral vectors encoding TCR genes.

**Figure 5:**
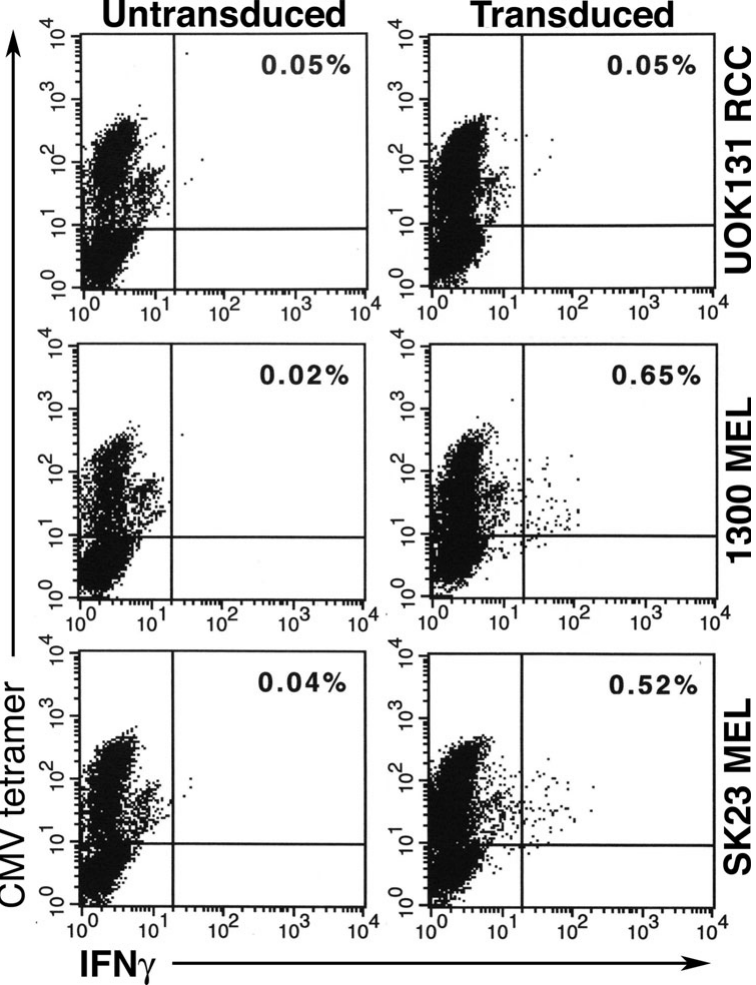
**Tumor cell recognition by CMV tetramer^+ ^T cells. **TIL 5 TCR-transduced and untransduced CMV-stimulated bulk cultures were cocultured in the presence of 10 μg/ml brefeldin A for 5 hours in a 1:1 ratio with A2^+^, MART^+ ^tumor cells (SK23 MEL, 1300 MEL) or A2^+^, MART-1^- ^tumor cells (UOK131 RCC). Cells were then collected, stained with PE-conjugated HLA-A2 MHC CMV tetramer, fixed, permeabilized, and stained with FITC-conjugated anti-IFN-γ mAb. Samples were analyzed using two-color flow cytometry. The percentage of dual positive staining cells (upper right quadrant) is as indicated.

Although the reactivity with MART-1:27–35 peptide loaded T2 cells shown in Figure 4 confirmed that we successfully engineered CMV pp65 peptide stimulated PBL-derived T cell cultures to contain bifunctional T cells, it was important to determine if these cultures could recognize the physiologic levels of antigen presented by tumor cells. When stimulated with HLA-A2^+ ^MART-1^+ ^tumor cells, 0.65% (1300 MEL) and 0.52% (SK23 MEL) of the T cells were HLA-A2/CMV pp65 tetramer positive and interferon-γ positive indicating that approximately 20% of the peptide reactive T cells were also tumor reactive (Figure 5). Tumor cells are poor antigen presenters relative to T2 cells because they often fail to express the accessory molecules required for efficient T cell recognition. Furthermore, only those T cells with sufficient TIL 5 TCR expression to yield high avidity T cells are capable of responding to the levels of processed antigen on the surface of tumor cells. This explains why a smaller fraction of bifunctional T cells are reactive with tumor cells compared to peptide-loaded T2 cells.

### MART-1 Recognition by a TIL 5 TCR-transduced gp100-reactive T cell clone

**Figure 6:**
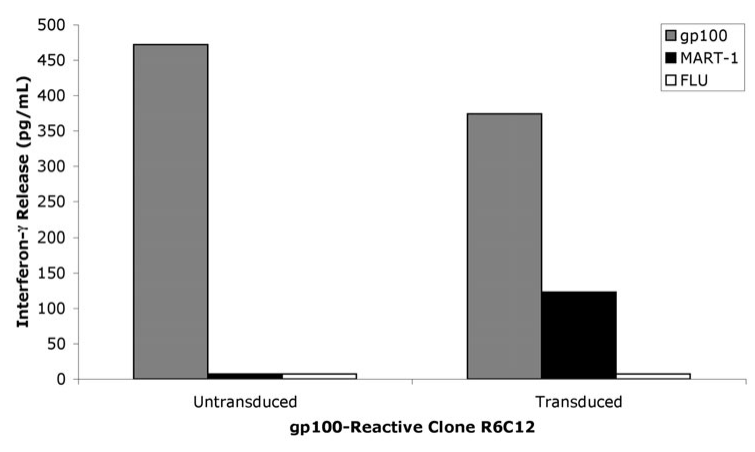
**Peptide recognition by TIL 5 TCR-transduced gp100-reactive T cell clone. **TIL 5 TCR-transduced and untransduced R6C12 cells were cocultured for 24 hours in a 1:1 ratio with T2 cells loaded with 5 μg/ml of peptide. Supernatants were collected and the amount of interferon-γ released was measured using ELISA. Values are the average of duplicate wells.

To demonstrate that the creation of bifunctional T cells capable of recognizing two tumor antigens was possible using our transduction methods, T cell clone R6C12 cells were activated with anti-CD3 and anti-CD28 mAb then transduced to express the TIL 5 TCR. TIL 5 TCR-transduced R6C12 cells were cocultured with gp100:209–217, Influenza M1, or MART-1:27–35 peptide-loaded T2 cells. As shown in Figure 6, TIL 5 TCR transduced R6C12 cells released interferon-γ when stimulated with MART-1:27–35 or gp100:209–217 loaded T2 cells, but not T2 cells loaded with the irrelevant influenza M1 peptide. In contrast, untransduced R6C12 cells only released interferon-γ when stimulated with gp100:209–217 loaded T 2 cells.

## Discussion

Here we report the successful engineering of T cells that are able to respond independently to two unrelated known antigens via both an endogenous and a retrovirally-transduced T cell receptor. These T cells were able to respond to low concentration of peptide, and were able to recognize antigen-positive tumor cells. By utilizing the initial antigen response as the activation for transduction, our 12-day protocol represents an efficient technique for generating bifunctional T cells from donor blood, and theoretically can be applied to any tumor or viral antigen in the context of one or more MHC restricting elements.

Many previous efforts at creating TCR transductants used non-specific activation of bulk or clonal populations [7,8,12] or, for the creation of bifunctional T cells, specific activation of semi-clonal populations with peptide-loaded autologous PBMC [5]. Non-specific T cell activation fails to expand T cell populations with known reactivity hence making it virtually impossible engineer T cells reactive with two know antigens. Engineering clonal or semi-clonal populations of T cells will create T cells reactive with two known antigens [5]. However, this process necessitates the establishment of antigen reactive T cell clones or long term T cell cultures prior to transduction. Although technically feasible, the creation of bifunctional T cells from T cell clones (this study) or long term T cell cultures [5] is time consuming and in our experience has a comparatively low yield of bifunctional cells. Furthermore, it is likely that the reactivity and therapeutic efficacy of T cells are diminished with extended culturing (13). Therefore, any method capable of rapidly producing bifunctional T cells will be better suited to clinical applications.

In contrast to using anti-CD3 mAb, *in vitro *stimulation with antigenic peptides will preferentially activate antigen reactive T cells to expand. These proliferating antigen reactive T cells can be transduced to express a second TCR. Based on our tetramer analysis, only 0.7% of the unstimulated donor PBL stained with the CMV tetramer (data not shown), compared to 44.6% of our peptide stimulated populations (data not shown). This profound expansion allowed for more efficient transduction, and 2.7% of the resulting culture was measurably bifunctional (figure 4). As retroviral transduction and *in vitro *selection for transduced T cells becomes more efficient, the frequency of bifunctional T cells in these cultures will increase to the point where it is feasible to treat patients.

The combination tumor/viral bifunctional cells we have generated here may have novel uses in immunotherapy, such as bypassing tumor- or viral-induced T cell unresponsiveness. Fossati and colleagues demonstrated that naïve bifunctional T cells "preactivated" via one TCR prior to adoptive transfer would then mediate cytotoxicity via the second TCR [14]. Animal and *in vitro *studies have shown that peripherally-induced tolerance can be reversed, resulting in regained T cell responsiveness [15,16]. It may be possible to reactivate tolerized T cells *in vitro *or *in vivo *by activating a second T cell receptor specific for a non-tolerized antigen [16,17]. In addition, viral antigens such as those associated with influenza, trigger alternate T cell activation pathways [18] and have been shown to elicit a strong T cell immune response [19]. Redirecting the vigorous anti-viral T cells which have not been exposed to the immunologic tolerance associated with most tumor-reactive T cells may be effective in eradicating tumor burden.

The substantial proliferation in response to strong immunogens such as viral antigens can also be used to improve the localization of T cells that also have anti-tumor activity. Using murine bifunctional T cells created by retroviral transfer of a chimeric immunoglobulin receptor specific for an ovarian cancer-associated tumor antigen to alloreactive T cells, Kershaw and colleagues were able to demonstrate *in vivo *expansion in response to alloimmunization and demonstrated anti-tumor activity [20]. It is possible that tumor/viral bifunctional cells would also behave in this way, and we are currently working on murine models with human/mouse chimeric TCRs to test this hypothesis.

In addition, some current immunotherapy protocols for the treatment of metastatic melanoma involve immunodepletion prior to adoptive cell transfer [21]. Such protocols are similar to solid organ and stem cell transplantation in that the patients are temporarily immunosuppressed and at risk for reactivation of latent viruses such as CMV and Epstein-Barr virus. Tumor/viral bifunctional T cells may be particularly useful in this setting, where the anti-viral activity may help treat reactivation, and the reactivation of the virus may further boost the anti-tumor activity of the T cells by inducing additional stimulation of the bifunctional cells.

Another consideration to bear in mind with the creation of bifunctional T cells is alternate pairing of the alpha and beta chains resulting in the combination of novel T cell receptors within a bifunctional cell. These T cells could have undesirable autoimmune properties. This could be circumvented by identifying T cells within a bifunctional population that have maximal expression of both the endogenous and introduced TCRs, indicating minimal cross-pairing of chains [6]. Screening for these T cells and selectively expanding them would reduce the risk of untoward autospecificity.

In our experiences, it has been difficult to transduce PBL-derived T cells from normal donors that are stimulated with antigenic peptides derived from self-antigens (data not shown). This is likely due to the low precursor frequency and/or the state of immunologic tolerance of T cells reactive with antigens such as gp100 or tyrosinase [20,22,23]. These limitations do not preclude generating T cells capable of recognizing two different tumor antigens, for we have demonstrated here that a T cell clone reactive with gp100:209–217 can be engineered to also recognize MART-1. However, transducing T cell clones is more time consuming since it is first necessary to isolate the T cells clones prior to transduction. There are two potential strategies for overcoming the limitations of transducing T cells with low precursor frequencies or that are immunologically tolerant. First, is transducing actively expanding tumor infiltrating lymphocyte cultures which contain tumor antigen-reactive T cells [24]. Second, patients vaccinated against tumor associated self antigens often have increased frequencies of antigen reactive T cells in their peripheral blood [23], and these T cells may lend themselves to activation and expansion *in vitro *to enable efficient retroviral transduction.

## Conclusion

The approach for generating bifunctional T cells we describe in this study may be feasible for viral infections and malignancies and may represent a powerful approach for those patients that otherwise would fail immunotherapy due to the accumulation antigen- or MHC-loss variants.

## Abbreviations

PBMC, peripheral blood mononuclear cells; TCR, T cell receptor; TAA, tumor-associated antigen; CMV, cytomegalovirus

## Competing interests

The authors declare that they have no competing interests.

## Authors' Contributions

AL designed the experiments, performed the transductions, carried out the cocultures and flow cytometry and prepared the manuscript. GC engineered the CMV minigene and the CMV-expressing COS cells, and edited the manuscript. MN conceived of the study, oversaw design and execution of the experiments, and finalized the manuscript.
